# Association between neutrophil percentage-to-albumin ratio and rheumatoid arthritis: a cross-sectional study from US population, 1999–2018

**DOI:** 10.3389/fimmu.2025.1596359

**Published:** 2025-07-11

**Authors:** Di Liu, Jing Zhang, Lan He, Qi An, Qianyun Xu, Shusen Zhang, Ruoyan Cao

**Affiliations:** ^1^ Department of Rheumatology and Immunology, The First Affiliated Hospital of Xi’an Jiaotong University, Xi’an, Shaanxi, China; ^2^ Department of Stomatology, Hunan University of Medicine, Huaihua, Hunan, China; ^3^ School and Hospital of Stomatology, Wenzhou Medical University, Wenzhou, Zhejiang, China

**Keywords:** rheumatoid arthritis, neutrophil-percentage-to-albumin ratio, risk factor, cross-sectional study, predictive model

## Abstract

**Background:**

Rheumatoid arthritis (RA) is linked to systemic inflammation and immune dysregulation. The neutrophil-percentage-to-albumin ratio (NPAR), integrating neutrophil activity and nutritional status, may reflect inflammatory and immune responses. However, its association with RA remains unexplored. We aimed to investigate the relationship between NPAR and RA using data from the National Health and Nutrition Examination Survey (NHANES) 1999–2018.

**Methods:**

This cross-sectional study utilized data from NHANES including 38,272 participants. The NPAR was calculated based on the neutrophil percentage (in total WBC count) (%), and albumin value (g/dL). We employed weighted multivariable logistic regression analysis and subgroup analysis to examine the association between NPAR and RA, adjusting for sociodemographic, lifestyle, and clinical covariates. Restricted cubic splines were used to assess potential non-linear relationships. Additionally, receiver operating characteristic (ROC) was performed to determine the predictive accuracy of NPAR compared with other inflammatory markers.

**Results:**

After adjusting for all covariates, multivariable logistic regression indicated that elevated levels of NPAR were significantly associated with an increased risk of RA (OR_tertile3vs1_ = 1.27, 95% CI: 1.11-1.44). A nonlinear, reverse L-shaped relationship was observed, with RA risk rising significantly when NPAR exceeded 13.6 (*P*_non-linearity_ = 0.004). Subgroup analyses confirmed consistency across populations. NPAR demonstrates a superior predictive capability for RA risk when compared to other established markers, including Systemic Immune-Inflammation Index (SII), Systemic Inflammation Response Index (SIRI), Platelet-to-Lymphocyte Ratio (PLR), Monocyte-to-Lymphocyte Ratio (MLR), and Neutrophil-to-Lymphocyte Ratio (NLR).

**Conclusion:**

Overall, our study demonstrates a significant positive association between NPAR and RA prevalence in U.S. adults, particularly when NPAR levels exceeded 13.6. Our findings underscore the critical role of immune-nutritional interactions in RA pathogenesis. However, owing to the cross-sectional design, prospective longitudinal investigations are warranted to establish causality and elucidate underlying biological mechanisms.

## Introduction

Rheumatoid arthritis (RA) is a systemic autoimmune disease characterized by chronic, progressive synovitis and joint destruction. The global prevalence of RA is estimated to range from 0.25% to 1% (1). Although RA can affect individuals of any age, those over 40 years are more susceptible to RA, leading to increased disability, reduced life expectancy, and significant socioeconomic challenges (2). The mechanisms underlying RA are complex and only partially understood. The onset of RA is influenced by interactions between genetic predispositions and environmental factors (3). Within the synovial microenvironment, stromal cells, immune cells, and various inflammatory mediators contribute to the maintenance of chronic inflammation and the promotion of joint damage (4–6). Given the rising prevalence of RA and its associated socioeconomic consequences, it is crucial to identify risk factors early to enable timely therapeutic interventions.

The neutrophil-percentage-to-albumin ratio (NPAR) serves as a novel and effective biomarker that combines the neutrophil percentage and albumin value to reflect systemic inflammation and immune conditions (7). Research indicates that NPAR can assess risk and prognosis across a variety of conditions (8–10). However, its association with RA has not yet been documented. Neutrophils, the most abundant leukocytes in both white blood cells and synovial fluid, are closely linked to inflammatory processes and the immune responses associated with RA (11). Serum albumin, a key marker reflecting a patient’s nutritional health, possesses antioxidant and anti-inflammatory properties. Research indicates that patients with RA often exhibit reduced serum albumin levels, which may result from several factors, including chronic inflammation and malnutrition related to the condition (12). Based on these findings, it is reasonable to speculate that NPAR may demonstrate a positive correlation with RA.

Given that NPAR is a readily accessible and cost-effective indicator of systemic inflammation and immune response, understanding and validating its predictive significance would greatly assist physicians in their clinical decision-making. This study aimed to evaluate NPAR levels in adults with RA in the United States, utilizing data from the National Health and Nutrition Examination Survey (NHANES), and to explore the association between NPAR and RA.

## Methods

### Study population

This cross-sectional study used ten cycles of survey data from NHANES 1999-2018. NHANES employs a stratified, multistage, clustered probability sampling methodology orchestrated by the National Center for Health Statistics (NCHS) to assess the health and nutritional status of the civilian population in the United States. Data collection for NHANES involved interviews, as well as laboratory and physical assessments, with each participant providing written informed consent. For the purposes of this study, individuals who were uncertain about having RA and those lacking data on neutrophil percentage and albumin were excluded based on the established criteria.

### Exposure variable

NPAR was calculated using the formula: [Neutrophil percentage (%)/Albumin concentration (g/dl)]. Blood sample analysis was conducted using the Beckman CoulterH 800 instrument at the NHANES mobile examination center. To assess albumin concentration, the NHANES Standard Biochemistry Profile was employed, utilizing a bichromatic digital endpoint method.

### Outcome variable

The outcome of this study was RA. RA was diagnosed by professionals and the information was collected through a questionnaire survey. Briefly, two questions related to arthritis were posed to the participants. Firstly, they were asked: “Has a doctor or other health professional ever told you that you have arthritis?” Those who answered “yes” were then asked a second question, “Which type of arthritis was it?” The participants who answered “rheumatoid arthritis” were included in the study.

### Covariates

Covariates included age (< 60 years old, ≥ 60 years old), sex (male or female), race/ethnicity (non-Hispanic white, others), education (< high school, high school, ≥ 60 high school), marital status (married/living as married, never married or separated/divorced/widowed), smoking status (non-smokers or smokers), obesity (non-obese: BMI < 30 or obese: BMI ≥ 30), diabetes, hypertension, and hyperlipidemia.

### Statistical analysis

In this research, the weights were considered in accordance with the NHANES analysis guidelines. Continuous variables were reported as mean and standard error (SE), while categorical variables were presented as percentages. To assess the baseline characteristics across different NPAR levels, Analysis of Variance (ANOVA) was employed for continuous variables and the chi-square test was utilized for categorical variables.

Weighted multivariable logistic models were applied to investigate the relationship between NPAR and RA accounting for potential confounding variables. Model I included adjustments for age, sex, and race, while model II incorporated additional factors such as marital status, annual household income, smoking status, obesity, diabetes, hypertension, and hyperlipidemia. The restricted cubic splines were used to explore the non-linear relationships. Furthermore, stratified and interaction analyses were conducted to assess the relationship between NPAR and RA. The area under the curve (AUC) of the receiver operating characteristic (ROC) was used to assess the predictive accuracy of NPAR and other inflammatory indicators in predicting the prevalence of RA. We employed a complete case analysis approach in our study due to the minimal amount of missing data. All statistical analyses were performed using R software (version 4.1.2) with a significance threshold set at 0.05 to determine statistical significance.

## Results

### Baseline characteristics of study populations

**Figure 1:**
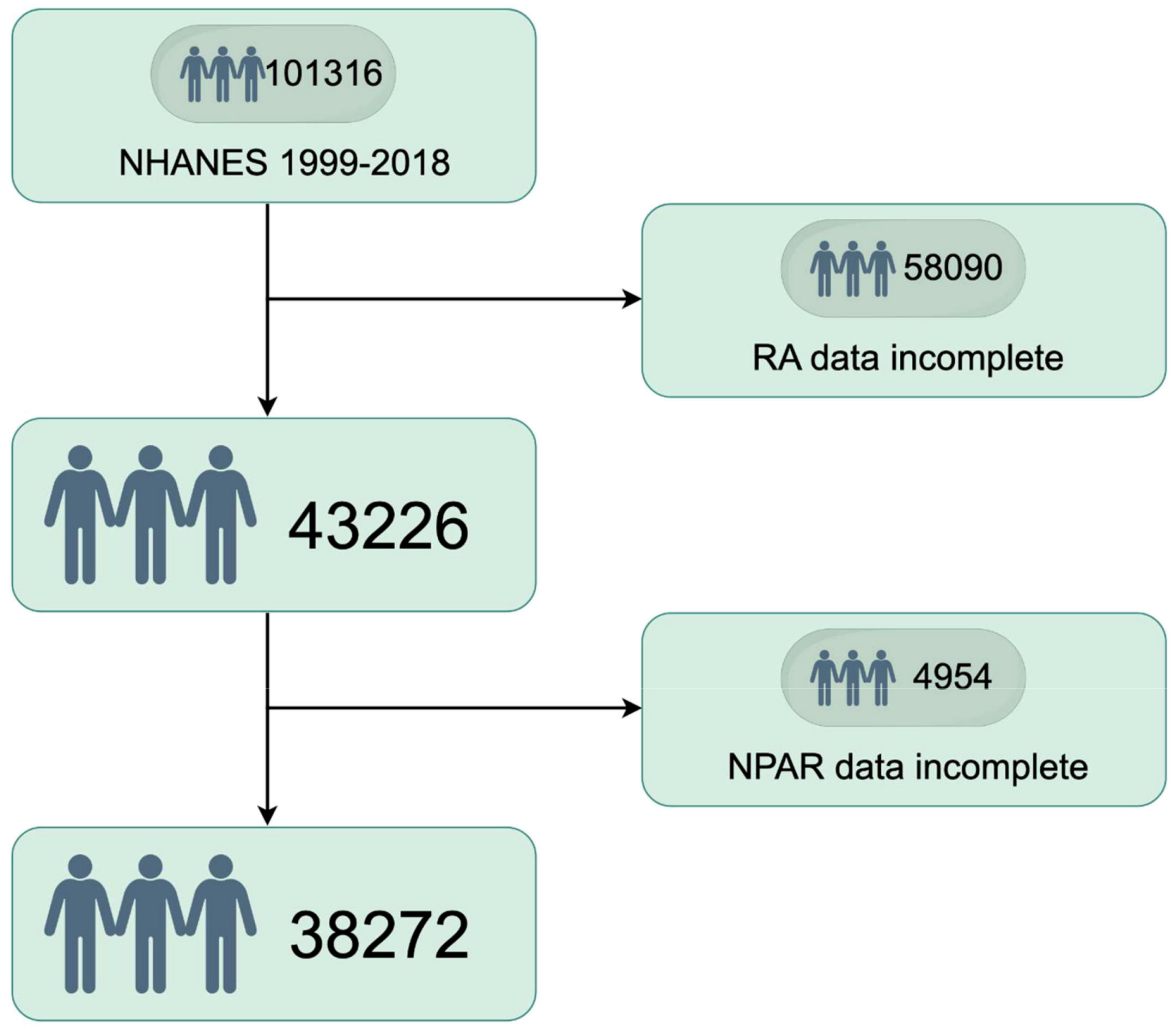
Study population selection (N=38,272) by Figdraw.

The process of selecting the study population, as illustrated in **Figure 1**, involved several stages during the NHANES 1999–2018 study. Participants with incomplete RA data (n = 58,090) and those with incomplete NPAR data (n = 4954) were excluded from the analysis. Ultimately, a total of 38,272 participants which represented approximately 161.8 million non-institutionalized residents of the United States were included in the study. The baseline characteristics of the study populations are summarized in **Table 1**. On average, the participants’ age was 43.74 years, with 50.59% male and 49.41% female. Significant differences were observed in age, age group, sex, race, education level, marital status, smoking habits, obesity rates, and the prevalence of diabetes, hyperlipidemia, and RA across various NPAR levels. The prevalence rates of RA among the different NPAR levels were 4.05%, 4.67% and 6.94%, respectively.

**Table 1:** Weighted characteristics of the participants (N=38,272).

Variable	NPAR				*P* value
	Overall	Tertile 1	Tertile 2	Tertile 3	
Age (year), mean (SE)	43.74 (0.17)	41.24 (0.23)	43.77 (0.21)	46.35 (0.21)	< 0.0001
Age group (%)					< 0.0001
≤ 60 years	83.71	87.88	84.61	78.28	
> 60 years	16.29	12.12	15.39	21.72	
Sex (%)					< 0.0001
Female	49.41	39.51	49.16	60.22	
Male	50.59	60.49	50.84	39.78	
Race (%)					< 0.0001
Non-Hispanic White	65.85	61.28	68.06	68.26	
Others	34.15	38.72	31.94	31.74	
Education^a^ (%)					< 0.0001
< High school	16.69	16.95	15.65	17.63	
High school	23.57	22.02	23.94	24.87	
> High school	59.64	61.03	60.41	57.5	
Marital status^a^ (%)					< 0.0001
Married/living as married	63.29	64.2	65.56	62.16	
Never married	19.82	23.28	18.81	18.04	
Separated/divorced/widowed	15.72	12.52	15.63	19.8	
Smoking status^a^ (%)					< 0.001
Non smoker	55.59	56.74	56.39	53.56	
Smoker	44.36	43.26	43.61	46.44	
Obesity^a^ (%)					< 0.0001
No	66.57	74.57	67.99	59.01	
Yes	32.2	25.43	32.01	40.99	
Diabetes mellitus^a^ (%)					< 0.0001
No	88.05	92.79	90.62	84.68	
Yes	10.28	7.21	9.38	15.32	
Hypertension ^a^ (%)					< 0.0001
No	69.08	73.28	69.83	63.82	
Yes	30.9	26.72	30.17	36.18	
Hyperlipidemia (%)					< 0.0001
No	31.56	34.82	30.98	28.76	
Yes	68.44	65.18	69.02	71.24	
Rheumatoid arthritis (%)					< 0.0001
No	94.82	95.95	95.33	93.06	
Yes	5.18	4.05	4.67	6.94	

a Missing values for total study: education (n =46, 0.12%), marital status (n = 381; 1%), smoking status (n = 29; 0.76%), obesity (n = 381, 1.50%), diabetes mellitus (n = 1271; 3.32%), hypertension (n = 10; 0.026%).

### Association between NPAR and RA

A weighted logistic regression analysis was conducted to investigate the association between NPAR and the risk of developing RA (**Table 2**). NPAR values were categorized into tertiles. The highest NPAR level was positively associated with an increased risk of developing RA in the crude model (OR = 1.77, 95% CI: 1.57-1.99), model I (OR = 1.41, 95% CI: 1.25–1.59), and model II (OR = 1.27, 95% CI: 1.11–1.44).

**Table 2:** Association between NPAR and RA.

Variable	Crude model		Model I		Model II	
	OR (95%CI)	*P*-value	OR (95%CI)	*P*-value	OR (95%CI)	*P*-value
NPAR (per SD)	1.30 (1.23, 1.37)	< 0.0001	1.20 (1.14, 1.26)	< 0.0001	1.18(1.12,1.25)	<0.0001
NPAR						
Tertile 1	Ref		Ref		Ref	
Tertile 2	1.16 (1.01, 1.34)	0.04	1.07 (0.92, 1.23)	0.39	1.01 (0.87, 1.17)	0.92
Tertile 3	1.77 (1.57, 1.99)	< 0.0001	1.41 (1.25, 1.59)	< 0.0001	1.27 (1.11, 1.44)	< 0.001
*P* for trend		< 0.0001		< 0.0001		< 0.001

Model I: Adjusted for age, sex, and race.

Model II: Model I and adjusted for education level, marital status, obesity, smoking status, diabetes mellitus, hypertension, and hyperlipidemia.

**Figure 2:**
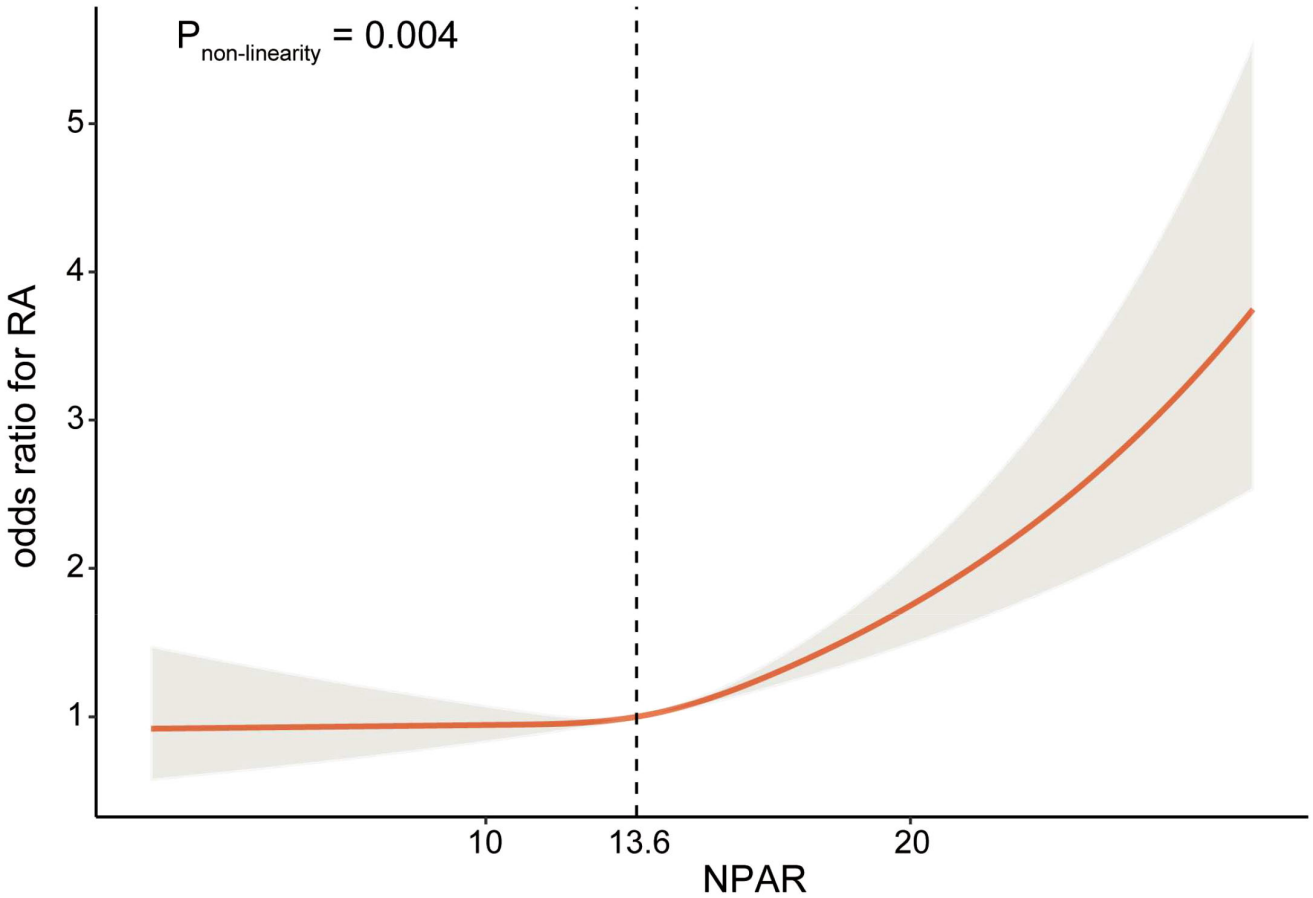
Relationship between NPAR and RA. Adjusted for age, sex, race, education level, marital status, obesity, smoking status, diabetes mellitus, hypertension, and hyperlipidemia.

Additionally, restricted cubic spline was employed to examine the non-linear relationship between NPAR and the risk of RA. The analysis revealed a significant reverse L-shaped association between NPAR and RA (*P*_non-linearity_ =0.004), as illustrated in **Figure 2**. When the NPAR levels were below 13.6, the risk of RA remained relatively stable; however, once NPAR levels exceeded 13.6, a significant increase in risk was observed.

### Subgroup analyses by potential effect modifiers

**Figure 3:**
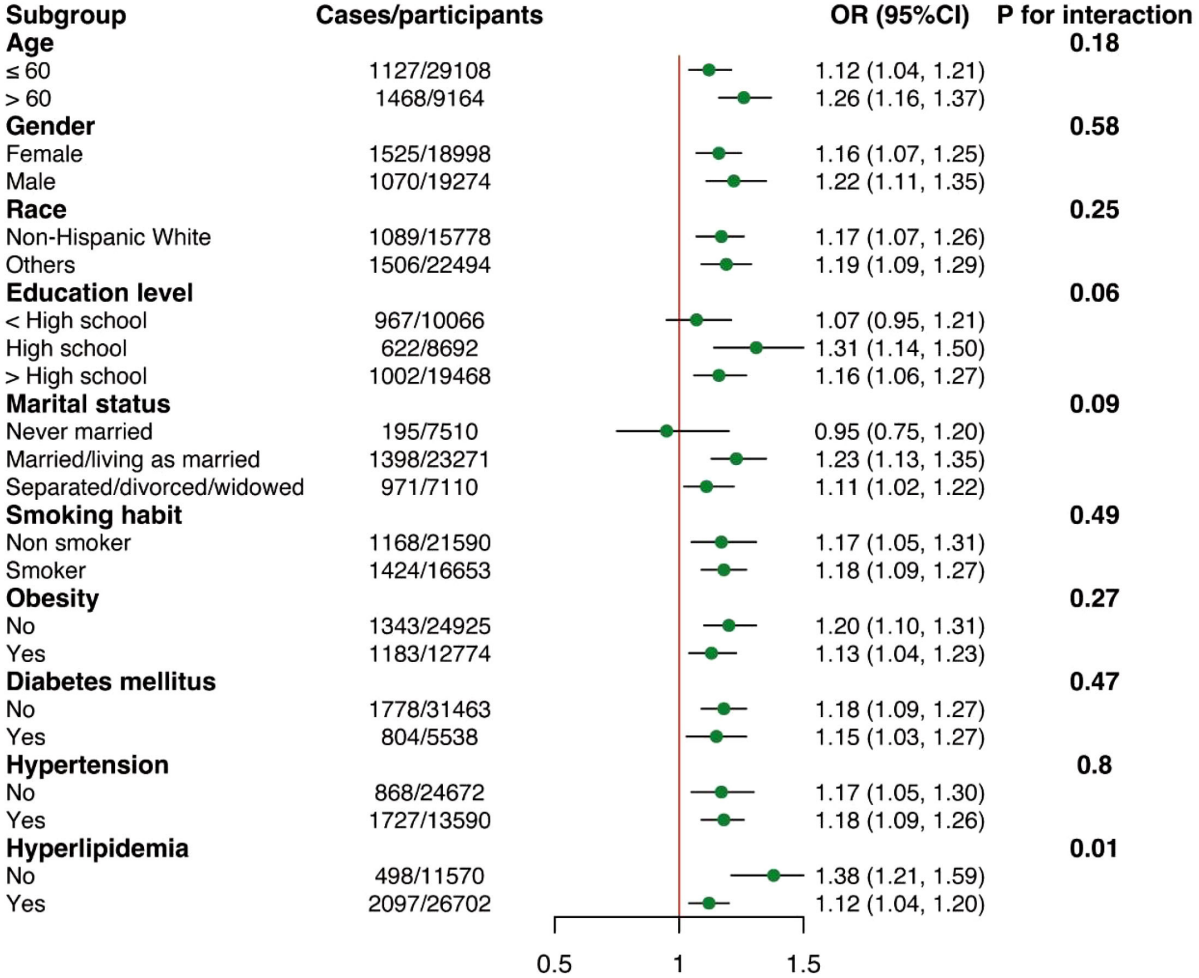
Subgroup analyses of the associations between NPAR and RA. Adjusted for age, sex, race, education level, marital status, obesity, smoking status, diabetes mellitus, hypertension, and hyperlipidemia except the subgroup variable.

The subgroup analyses regarding the association between NPAR and RA were displayed in **Figure 3**. Across all subgroups, elevated NPAR levels consistently showed an increased risk of developing RA. It was observed that hyperlipidemia might influence the relationship between NPAR and RA (*P*_-interaction_ = 0.01). Specifically, the risk of developing RA increased by 38% for each unit increase in NPAR in individuals without hyperlipidemia (OR = 1.38, 95% CI: 1.21-1.59), and by 12% for each unit increase in NPAR in individuals with hyperlipidemia (OR = 1.12, 95% CI: 1.04-1.20). However, as all the associations consistently showed the same direction, these results may not have significant clinical implications.

### Predictive accuracy of NPAR and other inflammatory indicators

**Figure 4:**
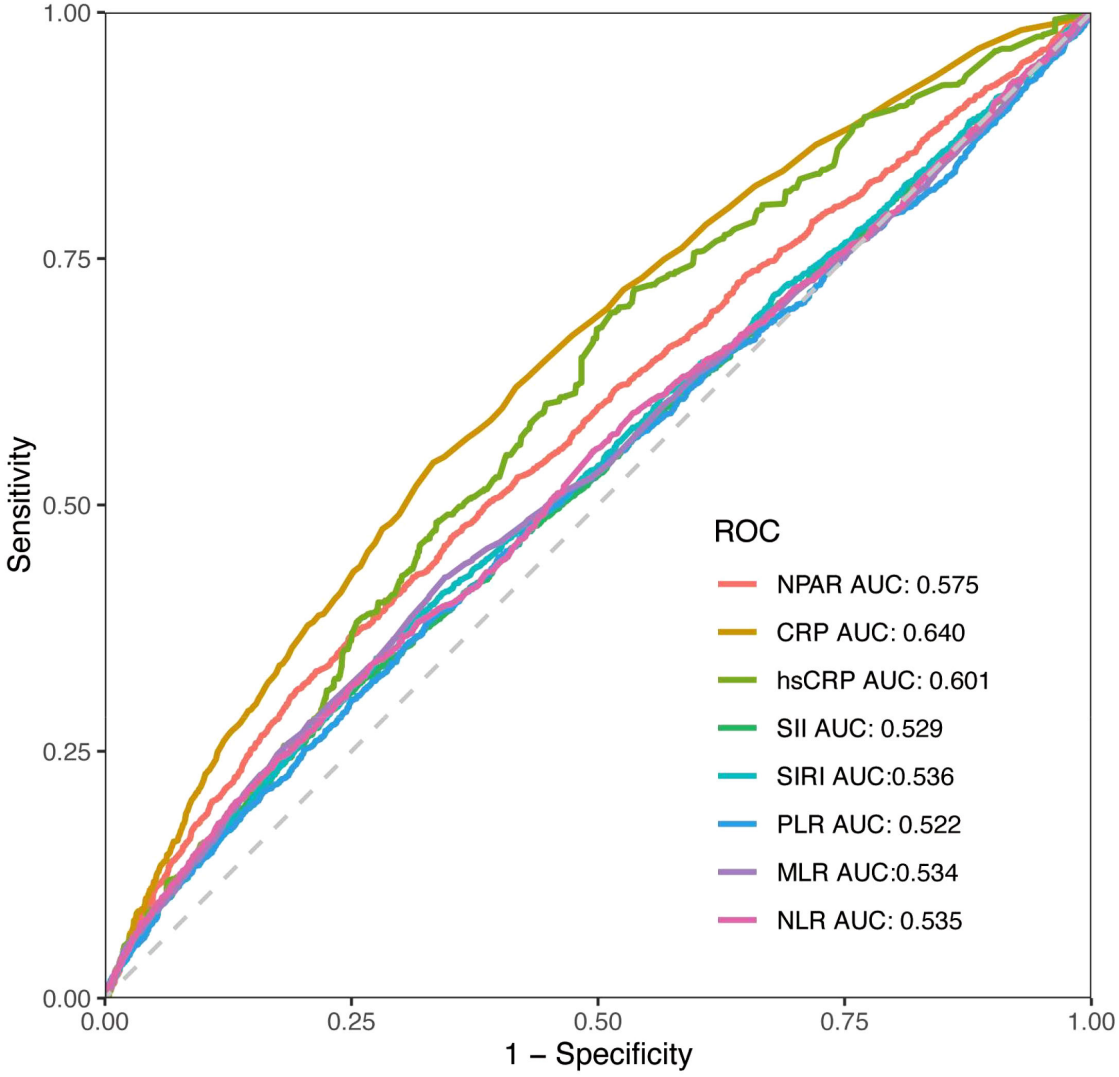
The ROC curves for displaying the predictive abilities of NPAR and other systemic inflammatory indicators.

ROC analyses were conducted to evaluate the discriminatory predictive performance of the NPAR in comparison to other inflammatory markers. The findings revealed that NPAR (AUC = 0.575) exhibited a greater predictive capacity than Systemic Immune-Inflammation Index (SII) (AUC = 0.529), Systemic Inflammation Response Index (SIRI) (AUC = 0.536), Platelet-to-Lymphocyte Ratio (PLR) (AUC = 0.522), Monocyte-to-Lymphocyte Ratio (MLR) (AUC = 0.534), and Neutrophil-to-Lymphocyte Ratio (NLR) (AUC = 0.535). Nevertheless, C-reactive protein (CRP) (AUC = 0.640) and high-sensitivity CRP (hsCRP) (AUC = 0.601) demonstrated superior predictive abilities (**Figure 4**).

## Discussion

In this study, we identified a notable correlation between NPAR and RA through a cross-sectional analysis of data from 38272 participants in NHANES 1999-2018. Additionally, the RCS analyses revealed a nonlinear positive correlation between NPAR and RA. When NPAR levels exceeded a certain threshold, the likelihood of developing RA increased significantly. Furthermore, this association was consistent across various subgroups. These findings underscored the importance of considering NPAR levels when assessing and managing RA.

The interplay between immune responses and inflammation is crucial for understanding the pathogenesis of RA, influencing both the severity and progression of the disease (2). NPAR, which encompasses the percentage of neutrophils and the concentration of albumin in the bloodstream, serves as an indicator of the body’s systemic immunological and inflammatory status, as well as its nutritional condition. Neutrophils play a vital role in the innate immune system and act as key effector cells in adaptive immunity and systemic inflammatory responses (13, 14). In the context of RA, neutrophils present in the blood exhibit a distinct activation profile characterized by the production of reactive oxygen species (ROS), secretion of cytokines, and delayed programmed cell death (apoptosis). Upon activation, these circulating neutrophils are rapidly recruited to areas of inflamed joints (11). The immunopathology driven by neutrophils is intricately associated with joint inflammation and the destruction of bone in RA (15). Moreover, neutrophils interact with various immune cells, further exacerbating the inflammatory loop in RA (16, 17). Activated neutrophils release neutrophil extracellular traps (NETs) in response to various stimuli. In RA, NETs enhance inflammation, directly promote bone erosion by stimulating osteoclast formation, and induce fibroblast-like synoviocyte (FLS) activation and cytokine release, thereby exacerbating joint damage and perpetuating a vicious cycle of NET induction and autoantibody formation (15, 18, 19). Furthermore, NETosis accelerates the modification and exposure of self-antigens in RA patients (20). In summary, neutrophils contribute to chronic inflammation, oxidative stress, and immune dysregulation in RA through multiple mechanisms, including ROS production, pro-inflammatory cytokine secretion, delayed apoptosis, and the release of NETs. Albumin, the most abundant protein in human plasma, plays a critical role in maintaining nutritional balance and osmotic pressure, in addition to exhibiting antioxidant and anti-inflammatory properties. Hypoalbuminemia is a prevalent condition in patients with RA (21). This condition exerts a detrimental effect on the disease progression of RA by impairing the body’s antioxidant defense mechanisms, thereby intensifying oxidative stress and promoting pro-inflammatory responses (22). Concurrently, inflammatory processes can exacerbate hypoalbuminemia through mechanisms such as inducing capillary leakage (23). The interplay between malnutrition and inflammation establishes a deleterious feedback loop, significantly influencing the trajectory of RA. Consequently, a higher NPAR reflects the interplay between neutrophil activity and albumin levels, contributing to the onset and progression of RA.

NPAR has emerged as a significant biomarker for assessing the risk and prognosis of various diseases, including cancer, kidney injury, cardiogenic shock, myocardial infarction, sepsis, depression and liver fibrosis (9, 24–28). Among patients undergoing peritoneal dialysis, elevated levels of NPAR were significantly associated with increased rates of all-cause mortality as well as cardio-cerebrovascular mortality, independent of other clinical factors. Moreover, NPAR outperformed other inflammatory markers such as CRP, NLR, and PLR in predicting adverse outcomes in this population (24). In the context of depression, a previous study identified a notable positive correlation between NPAR levels and depressive symptoms (29). Within the realm of autoimmune diseases, NPAR has shown promise as a predictor of disease severity and short-term outcomes in anti-NMDAR encephalitis (30). Currently, there is a lack of research regarding the relationship between the NPAR and RA. This study aims to fill this gap by providing new evidence that underscores the importance of NPAR as a risk factor for RA.

Unlike acute phase reactants (e.g., CRP) or cellular ratios (NLR/PLR), NPAR uniquely integrates hepatic synthetic function via albumin with innate immune activity through neutrophil percentage. This dual-axis approach potentially offers a unique perspective on the complex interplay between inflammation, immune and systemic homeostasis, providing a more comprehensive assessment. Our ROC results indicate that NPAR, despite not surpassing CRP/hsCRP, possesses greater discriminatory ability than several commonly referenced inflammatory ratios for predicting RA. This positions NPAR as a clinically accessible and cost-effective adjunctive tool derived from routine blood work. Future research should explore whether combining NPAR with CRP/hsCRP or other clinical parameters could further enhance predictive models for arthritis outcomes.

Our research identified an optimal cutoff for NPAR at which the correlation between NPAR and RA changes. Specifically, when NPAR exceeds 13.6, a positive association emerges between NPAR and RA, indicating that an increase of one unit in NPAR is associated with a rise in RA prevalence. This nonlinear relationship may reflect the varying effects of different NPAR levels on RA prevalence. These findings suggest that NPAR could serve as a promising predictive biomarker for RA, potentially providing a new pathway for early detection and intervention. Further large-scale prospective studies are necessary to validate these results and to investigate the mechanisms underlying this relationship.

Our study has several notable strengths. To our knowledge, it was the first study to investigate the relationship between NPAR and RA. Additionally, the analyses were conducted on a large sample size, which enhances the validity of our findings. However, it is important to acknowledge several limitations. First, the cross-sectional design of the study restricts the ability to establish a causal relationship between NPAR and RA. Second, to verify a potential causal connection, future prospective multicenter studies will be essential., assessing the neutrophil percentages and albumin levels at a single baseline time point may not accurately reflect the chronic inflammation, immune, and nutritional status over time. Furthermore, the exclusion of unmeasured confounding variables limits the capacity to address potential residual confounding effects. Lastly, RA diagnosis in NHANES relies on self-reported data, which may introduce recall bias and misclassification. The data also lack of information confirming the stage of RA advancement. Nevertheless, validation studies support its utility in large-scale epidemiological research (31, 32). To mitigate potential biases, we adjusted for covariates influencing self-reporting accuracy (e.g., age, education level) through multivariable analyses. Future studies should employ multi-source validation (e.g., medical records, serological testing) to enhance RA phenotyping precision.

## Conclusion

In conclusion, our findings observed a nonlinear positive correlation between NPAR and RA in adults in the United States, with an inflection point of 13.6. Our findings underscore the critical role of immune-nutritional interactions in RA pathogenesis. However, owing to the cross-sectional design, prospective longitudinal investigations are warranted to establish causality and elucidate underlying biological mechanisms.

## Data Availability

The original contributions presented in the study are included in the article/supplementary material. Further inquiries can be directed to the corresponding authors.
